# Supplementary material for: Perturbation-Based Modeling Unveils the Autophagic Modulation of Chemosensitivity and Immunogenicity in Breast Cancer Cells
Source: Metabolites. 2021 Sep 18;11(9):637. doi: 10.3390/metabo11090637 (PMC8469554; doi:10.3390/metabo11090637)
Supplement: Supplementary file 1 [file metabolites-11-00637-s001.zip › SupplementaryFiles/Supplementary figures.pdf]

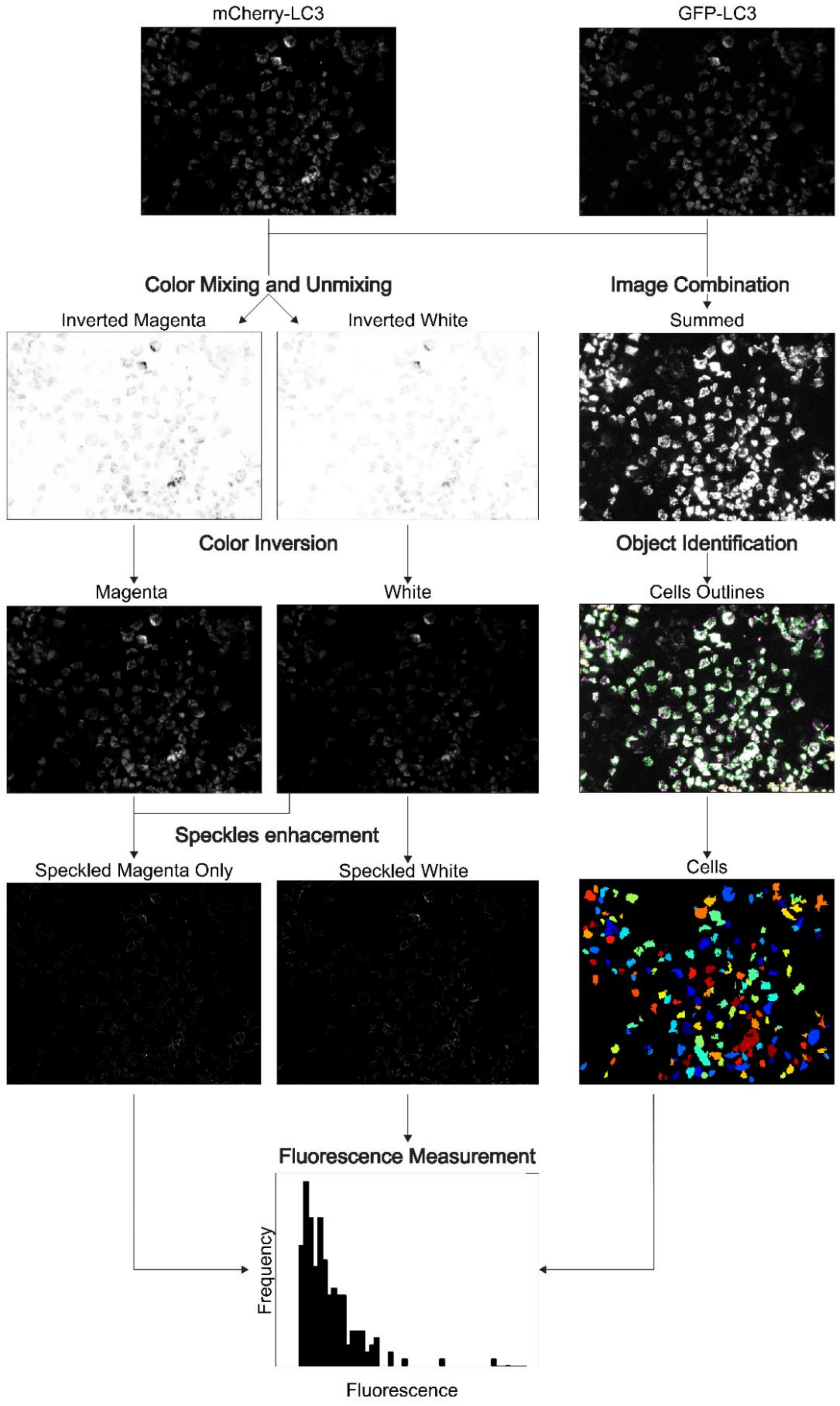

Figure S1. Image analysis pipeline.

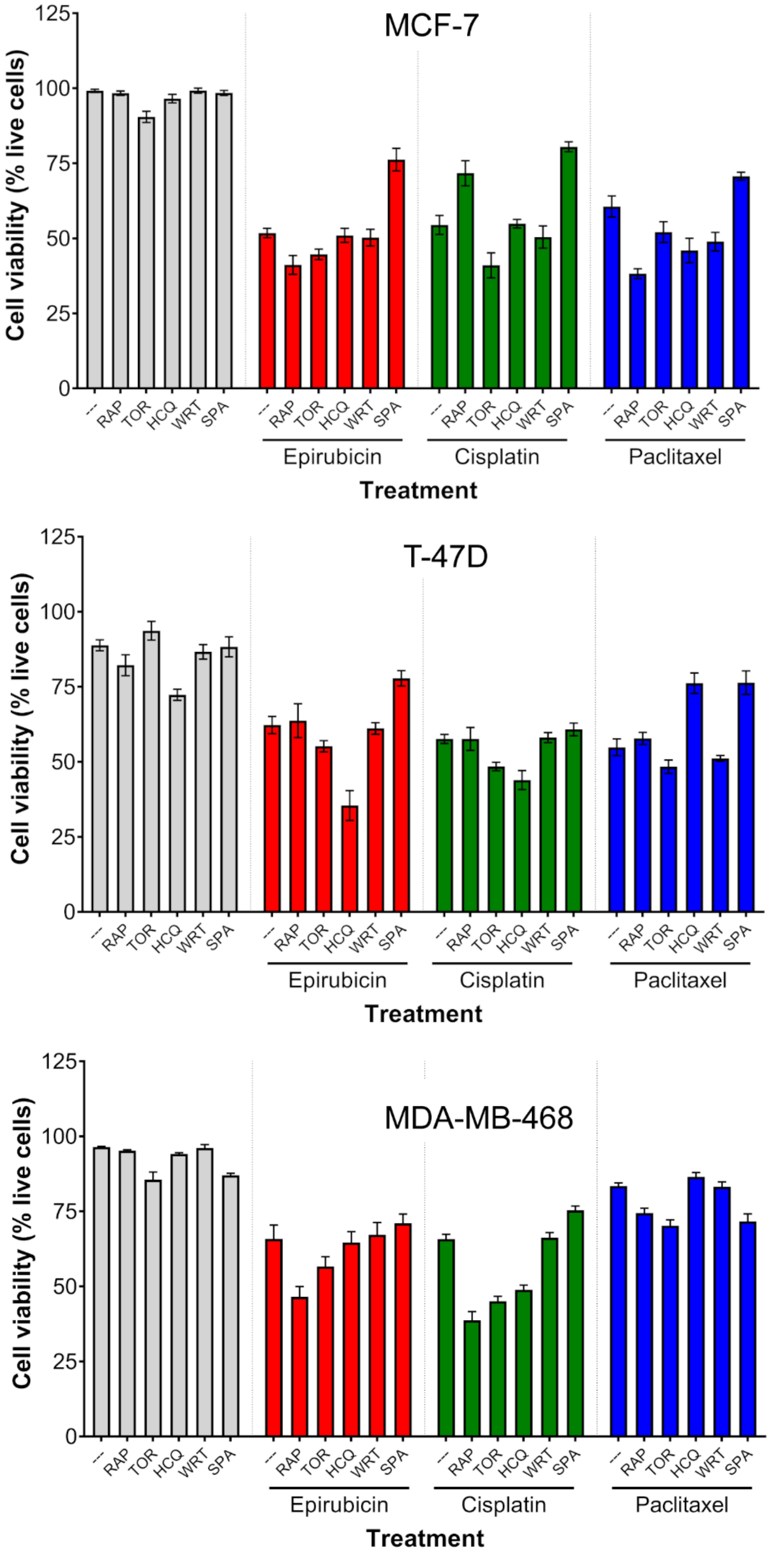

Figure S2. Cytotoxicity profiles for each chemotherapeutic drug and autophagy regulator combination for each cell line. CIS: cisplatin, EPI: epirubicin, H or HCQ: hydroxychloroquine, PAC: paclitaxel R: rapamycin, S: Spautin-1, T: Torin-2, W: wortmannin.

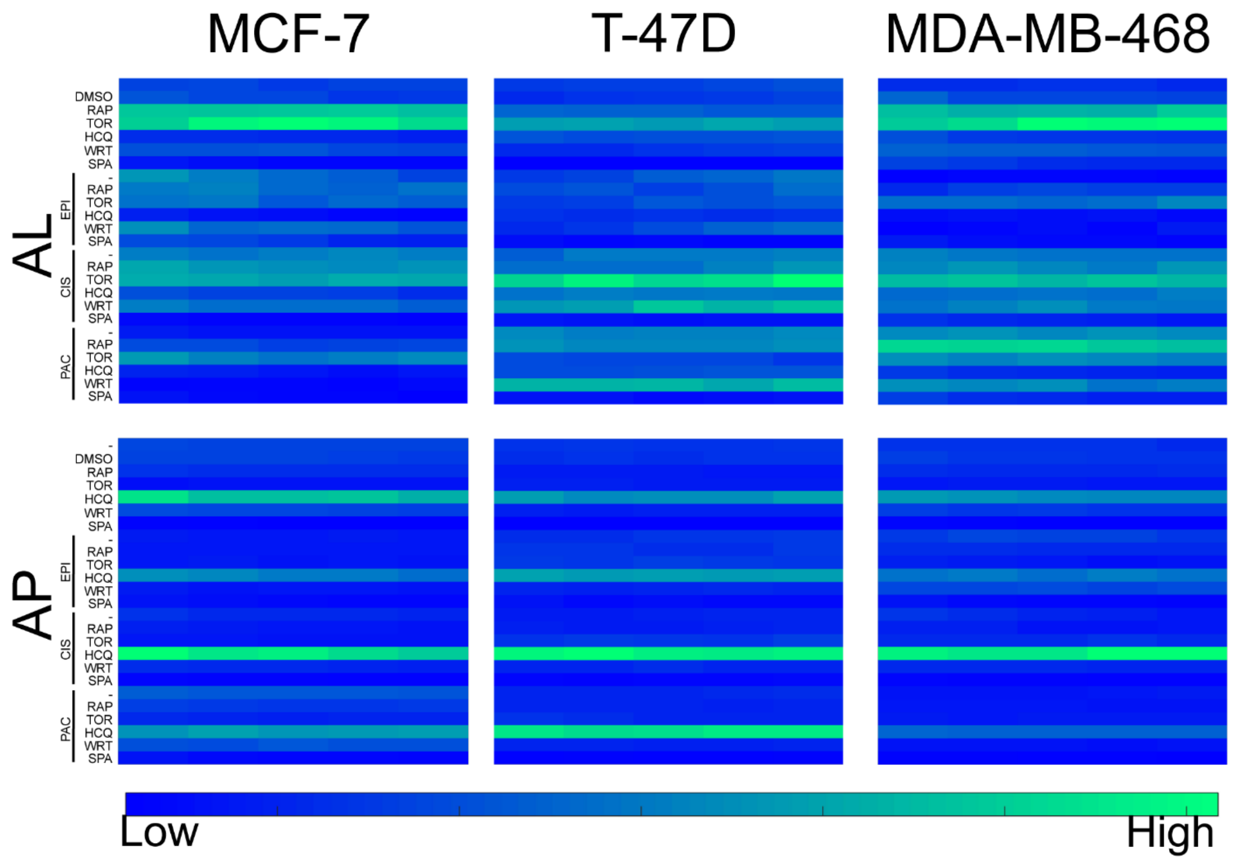

Figure S3. Dynamic changes in AL and AP composition of the autophagy sensor in three breast cancer cell lines upon multiple drug combinations. AL: autophagolysosomes, AP: autophagosomes, CIS: cisplatin, CYT: cytoplasm, DMSO: dimethylsulfoxide, EPI: epirubicin, HCQ: hydroxychloroquine, PAC: paclitaxel RAP: rapamycin, SPA: Spautin-1, TOR: Torin-2, WRT: wortmannin.
